# Targeting neoantigens to APC-surface molecules improves the immunogenicity and anti-tumor efficacy of a DNA cancer vaccine

**DOI:** 10.3389/fimmu.2023.1234912

**Published:** 2023-08-29

**Authors:** Marina Barrio-Calvo, Søren Vester Kofoed, Sofie Cens Holste, Anders Bundgård Sørensen, Nadia Viborg, Jens Vindahl Kringelum, Daniela Kleine-Kohlbrecher, Christian Skjødt Steenmans, Christian Bahne Thygesen, Birgitte Rønø, Stine Friis

**Affiliations:** Evaxion Biotech, Hørsholm, Denmark

**Keywords:** APC-targeting, CCL19, neoantigens, DNA vaccine, cancer immunotherapy

## Abstract

**Introduction:**

Tumor-specific mutations generate neoepitopes unique to the cancer that can be recognized by the immune system, making them appealing targets for therapeutic cancer vaccines. Since the vast majority of tumor mutations are patient-specific, it is crucial for cancer vaccine designs to be compatible with individualized treatment strategies. Plasmid DNA vaccines have substantiated the immunogenicity and tumor eradication capacity of cancer neoepitopes in preclinical models. Moreover, early clinical trials evaluating personalized neoepitope vaccines have indicated favorable safety profiles and demonstrated their ability to elicit specific immune responses toward the vaccine neoepitopes.

**Methods:**

By fusing *in silico* predicted neoepitopes to molecules with affinity for receptors on the surface of APCs, such as chemokine (C-C motif) ligand 19 (CCL19), we designed an APC-targeting cancer vaccine and evaluated their ability to induce T-cell responses and anti-tumor efficacy in the BALB/c syngeneic preclinical tumor model.

**Results:**

In this study, we demonstrate how the addition of an antigen-presenting cell (APC) binding molecule to DNA-encoded cancer neoepitopes improves neoepitope-specific T-cell responses and the anti-tumor efficacy of plasmid DNA vaccines. Dose-response evaluation and longitudinal analysis of neoepitope-specific T-cell responses indicate that combining APC-binding molecules with the delivery of personalized tumor antigens holds the potential to improve the clinical efficacy of therapeutic DNA cancer vaccines.

**Discussion:**

Our findings indicate the potential of the APC-targeting strategy to enhance personalized DNA cancer vaccines while acknowledging the need for further research to investigate its molecular mechanism of action and to translate the preclinical results into effective treatments for cancer patients.

## 1 Introduction

Therapeutic cancer vaccines (TCVs) utilize tumor antigens to elicit immune recognition of malignant cells, followed by controlled elimination of cancers (1).

A novel type of tumor-specific antigens is neoepitopes. Neoepitopes originate from non-synonymous mutations in the tumor genome and result in small, mutated peptides presented on major histocompatibility complex (MHC) molecules exclusively on the surface of tumor cells. When included in a TCV, neoepitopes hold the potential to generate effector T cells for the specific elimination of tumor cells while avoiding potential unwanted damage to healthy tissues. Mutations in the tumor genome are often unique to each individual patient, and identifying clinically relevant neoepitopes across patients is, therefore, challenging. Personalized approaches that utilize patient-specific neoepitopes for developing TCVs can be advantageous in overcoming this challenge (2).

Neoepitope-based TCVs have shown therapeutic efficacy in preclinical models with different vaccine delivery platforms, and more recently, they have achieved robust tumor-specific immune responses in the clinic (2–5). Among them, plasmid DNA (pDNA) is an attractive delivery platform for developing neoepitope-based TCVs as it is simple, generally considered safe, and versatile (6, 7). Nevertheless, pDNA is less immunogenic than other nucleotide-based delivery platforms such as mRNA. Therefore, there is still a need to enhance the ability of DNA-based vaccines to induce robust and long-lasting effector T-cell responses, especially towards weaker antigens such as cancer neoepitopes (8).

Strategies to enhance the immunogenicity of pDNA vaccines include new backbone designs containing immunostimulatory sequences, co-administration of genetic adjuvants, or facilitated delivery by gene guns or electroporation (9). Targeting antigens to the surface of APCs is another successful strategy to amplify the magnitude of humoral and cellular responses induced by pDNA-delivered epitopes in preclinical settings (9–21). APC-targeting pDNA vaccines encode fusion proteins consisting of an antigen fused to a molecule with the ability to bind receptors on the surface of professional APCs, hereon called APC-binding molecule. The interaction between the APC-binding molecule and its receptors is hypothesize to facilitate active antigen internalization, increasing the chances of presentation on MHC molecules (22–25). Most APC-targeting vaccines direct the antigens to receptors on the surface of conventional dendritic cells (cDCs), which play a pivotal role in initiating anti-tumor immunity by priming cytotoxic CD8+ T cells. Of particular interest are the receptors involved in the endocytosis of extracellular antigens, such as DEC205 or Clec9a (14, 17, 26) and chemokine receptors (27–29). In addition to targeting antigens to the surface of APCs, chemokines expressed at the site of pDNA vaccination can act as genetic adjuvants mediating the proliferation, differentiation, and maturation of cDCs (30).

Although APC-targeting is a well-characterized strategy for delivering antigens of viral origin (17–19), less is understood about its effectiveness when delivering cancer antigens (13, 14, 25, 31). Furthermore, it is still under investigation whether APC-targeting strategies can enhance the immunogenicity and therapeutic effect of cancer neoepitopes (32).

In this study, we evaluated the efficacy of the APC-targeting strategy for delivering *in silico* predicted neoepitopes in a pDNA cancer vaccine formulation. More specifically, we assessed the effect of different APC-binding molecules on their ability to elicit neoepitope-specific CD8+ and CD4+ T-cell responses and to improve anti-tumor efficacy. Based on these readouts, we selected CCL19 for further studies. We investigated the importance of conjugating the neoepitopes to CCL19 by a covalent link and found it to contribute to a more rapid onset of the cellular immune responses. We showed a positive correlation between the dose of the CCL19-based APC-targeting TCV and its anti-tumor efficacy, as well as showing the longevity of the neoepitope-specific T cells. Finally, we show that administration of the CCL19-based APC-targeting TCV post-tumor inoculation, can achieve tumor control as a monotherapy.

## 2 Materials and methods

### 2.1 Design of neoepitope encoding pDNA vaccines

The DNA plasmids designed here were based on previously published vectors encoding five or thirteen 27-mer CT26 *in silico* predicted neoepitopes from the mouse tumor cell line CT26, linked by (Glycine-Serine (GS))_5_ as poly-epitopes on a string, called Neo5 and Neo13. The sequence and selection strategies of the neoepitopes used for these studies have been previously described by Viborg et al. (33).

The APC-targeting pDNA constructs were based on the above. The APC-targeting DNA inserts contained i) an APC-binding molecule, ii) a dimerization module consisting of hinge 1, hinge 4, and the CH3 domain (h1h4CH3) from human IgG3 (21), and iii) the poly-neoepitope unit consisting of five or thirteen neoepitopes. Each of the modules was fused by Glycine-Leucine (GL) linkers. The sequences of the APC-binding molecules chemokine (C-C motif) ligands 3, 4, 5, 19, 20, 21 (CCL3, CCL4, CCL5, CCL19, CCL20, CCL21), XCL1, GM-CSF, Fv αDEC205, Fv αClec9a, and Celc9a ligand are found in **Supplementary Table 1**. In the absence of an endogenous secretion signal in the APC-binding molecule (Fv αDEC205, Fv αClec9a, and Clec9a ligand), the secretion signal of murine serum albumin (MKWVTFLLLLFVSGSAFS) was inserted in frame upstream. To design a non-targeted pDNA construct (NT_Neo5) encoding a secretion fusion protein unable to target any receptor, we introduced the secretion signal peptide from CCL3 upstream of the dimerization module.

To study the implications of the dimerization module in the immunogenicity of the delivered neoepitopes, we designed two additional constructs where the sequence of h1h4CH3 was either eliminated to design a monomeric APC-targeting DNA construct (CCL19_Neo5 monomer) or substituted with the dimerization sequence MHD2 from human IgM (CCL19_hMHD2_Neo5) (34).

All DNA insert sequences were optimized for codon adaptation in mice, GC content, repeated sequences, and mRNA-free energy using previously described tools (33). The DNA inserts were synthesized and subcloned into the expression vectors pUMVC4a (Aldevron, #4038) or pTVG4 (35) using EcoRI and NotI sites and upscaled by Aldevron (US) as *in vivo*-grade DNA.

To strengthen the translatability of the pDNA designs, empty pTVG4, and CCL19_Neo13 DNA plasmids were upscaled at Cobra Biologics (UK) as clinical-grade DNA.

### 2.2 Cell lines

BALB/c syngeneic colon cancer cell line CT26 (ATCC, #CRL2638) was cultured in RPMI (Gibco, #72400-021) supplemented with 10% heat-inactivated fetal calf serum (FCS) (Gibco, #10500-064). HEK293T cells (ATCC, #CRL-1573) were cultured in DMEM (Merk, #D6546) supplemented with 10% FCS, 1% GlutaMAX (ThermoFisher, #35050061), and 1% Penicillin/Streptavidin. CHO-K1 cells (ATCC, #CCL-61) were cultured in RPMI supplemented with 10% FCS and 1% Penicillin/Streptavidin. All cell lines were kept at 37°C and 5% CO_2,_ following the supplier’s instructions. All cell lines were regularly tested for mycoplasma.

### 2.3 DNA transfection

Adherent HEK293T or CHO-K1 cells were seeded in 6-well plates (0.25 x 10^6^ cells/well) and transfected after 48h using Lipofectamine 2000 (ThermoFisher, #L3000015). According to the manufacturer’s instructions, 1 µg of DNA plasmid and 6 µL of Lipofectamine were diluted in 125 µL of OptiMEM (ThermoFisher, #31985062) and added to each well. The supernatants were collected 48 hours after for analysis.

### 2.4 Immunoblotting

To evaluate the correct molecular weight of the different fusion proteins, the supernatant of transfected HEK293T cells was run with β-mercaptoethanol (Sigma-Aldrich, #63689) on a 4-20% Mini-Protean® TGX Stain-Free™ Protein Gel (BioRad, #4568093). After transfer to PVDF membranes (ThermoFisher, #88518), the membranes were blocked with 1x Pierce™ Clear Milk Blocking Buffer (ThermoFisher, #37587) and incubated overnight with Mouse anti-Human IgG (CH3 domain) Secondary Antibody (Invitrogen, #MA5-16557). After washing with TBS-Tween (ThermoFisher, #28360), the membranes were incubated for 45 min with Anti-mouse IgG HRP-conjugated (R&D systems, #HAF007) and developed using SuperSignal™ West Atto Ultimate Sensitivity Substrate (ThermoFisher, #A38555). Images were obtained using the iBright FL1500 Imaging System (Invitrogen, #A44241) and analyzed with iBright Analysis Software Version 5.1.0 (Invitrogen).

### 2.5 CCL19 sandwich ELISA

The expression levels of CCL19_Neo5 monomer, CCL19_Neo5, and CCL19_hMHD2_Neo5 were assessed in the supernatant of transfected CHO-K1 cells by murine CCL19 DuoSet sandwich ELISA (R&D systems, #DY440). CCL19/MIP-3 beta antibody pairs were used to capture and detect the fusion proteins. Plates were developed using DuoSet ELISA Ancillary Reagent Kit 2 (R&D Systems, #DY008) according to the manufacturer’s instructions.

### 2.6 DNA Immunizations

*Mice:* 6 to 8 weeks old BALB/c JrJ females were acquired from Janvier Labs (France). All animal experiments were conducted under the license 2017-15- 0201-01209 from the Danish Animal Experiments Inspectorate in accordance with the Danish Animal Experimentation Act (BEK nr. 12 of 7/01/2016), which is compliant with the European directive (2010/63/EU).

#### 2.6.1 Poloxamer formulation

Research and clinical grade pDNA were formulated with the co-block polymer poloxamer 188 as described by Viborg et al. (33). Mice received five intramuscular (i.m.) immunizations with one-week intervals in the left and right tibialis anterior muscles for a final volume of 100 μL per immunization unless otherwise indicated.

#### 2.6.2 Electroporation

Clinical grade pDNA was formulated in PBS and delivered via electroporation (EP) (AgilePulse, BTX, Harvard) immediately after i.m. injection. For therapeutic studies, mice received five i.m. immunizations spaced three to four days alternating in left and right tibialis anterior muscles for a final volume of 50 μL per immunization unless otherwise indicated.

### 2.7 Tumor challenge

Tumor challenges were conducted as previously described (33). Briefly, on the day of tumor cell inoculation (defined as study day 0), 5 x 10^5^ or 2.5 x 10^5^ CT26 cells were injected subcutaneously (s.c.) in the right flank of mice. The tumor volume was measured three times per week and calculated using the following formula: tumor volume = $\frac{\text{π}}{6}*{\left({\text{d}}_{1}{\text{*d}}_{2}\right)}^{3/2}$ . Experiments were terminated when the majority of tumors in the control groups reached a 12 mm diameter in any direction. Individual mice were euthanized upon reaching humane endpoints (i.e.15% loss of body weight or tumor ulcerations). When depicting longitudinal tumor volumes, missing data was mitigated by applying Last-Observation-Carried-Forward (LOCF).

### 2.8 MHC I multimer staining for detection of neoepitope-specific CD8+ T cells

The induction of neoepitope-specific CD8+ T cells was monitored during the animal studies as described previously (33). In brief, tail-vein blood from a representative number of mice was stained with fluorochrome-labeled antibodies to allow for the identification of CD8+ T cells, and fluorochrome-labeled MHC class I neoepitope-specific tetramers (murine allele H-2K^d^ loaded with C1 minimal peptide KFKASRASI, from hereon: C1 multimer) purchased from Tetramer Shop (Denmark). The full gating strategy is illustrated in **Supplementary Figure 7A**.

### 2.9 Spleen isolation

Upon termination of the studies, spleens from a representative number of mice were harvested and collected in RPMI supplemented with 10% FCS (Gibco, #10500-064), processed into single cells suspensions by GentleMACS processing (Miltenyi Biotec, C-tubes, #130-096-334 and Dissociator, #130-093-235) and cryopreserved.

### 2.10 Peptide re-stimulation and intracellular cytokine staining or enzyme-linked immunosorbent spot

To detect neoepitope-reactive T cells induced by the vaccination, splenocytes were re-stimulated with synthetic neopeptides and analyzed by intracellular cytokine staining (ICS) or enzyme-linked immunosorbent spot (ELISpot).

*Synthetic neopeptides:* 27-mer peptides, corresponding to the neoepitopes encoded in the pDNA constructs, were synthesized by GenScript (New Jersey, USA). The 27-mer peptides feature the mutated AA in the central position. The lyophilized peptides were dissolved in dimethyl sulfoxide (DMSO) (Merck, #D8418) at 10 mg/mL for peptide re-stimulation.

*ICS:* 2 x 10^6^ splenocytes per well were stimulated with synthetic neopeptide pools corresponding to the vaccine content. Following re-stimulation, frequencies of interferon γ (IFNγ) and tumor necrosis factor α(TNFα)-producing CD4+ and CD8+ T cells were analyzed by flow cytometry and quantified as previously described (33). The full gating strategy is illustrated in **Supplementary Figure 7B**. Samples where less than 500 CD8+ T cells were acquired were excluded from the analysis.

*ELISpot:* 0.5 x 10^6^ splenocytes per well were plated in anti-IFNγ antibody-coated ELISpot plates and stimulated either with synthetic neopeptide-pools corresponding to the vaccine content or with the individual peptides overnight. IFNγ-positive cell spots were developed as previously described (33). The plates were imaged using a CTL ELISPOT Analyzer.

### 2.11 Ranking of APC-binding molecules

To evaluate the performance of the different APC-binding molecules, the pDNA vaccines were ranked according to i) anti-tumor efficacy, ii) induction of reactive CD8+ T cells, and iii) induction of reactive CD4+ T cells in the spleen compartment measured by ICS. The readouts for three features were normalized, scaling the maximum values to 1, and averaged to generate a score that is used to rank the efficacy of the APC-binding modules in the immunogenicity and anti-tumor efficacy of neoepitope-encoding pDNA designs.

### 2.12 Statistical analyses

GraphPad Prism 9 for Mac OS X was used for graphs and statistical analyses. Data were subjected to the Shapiro-Wilk test for normality (alpha = 0.05). Parametric data were analyzed by One-way ANOVA with Šidák´s test for multiple comparisons. Non-parametric data were analyzed by the Kruskal-Wallis test with Dunn’s multiple comparison correction. For the tests described above, the following levels of statistical significance are applied: ns p ≥ 0.05, *p< 0.05, **p< 0.01, ***p< 0.001, ****p< 0.0001. Kaplan-Meier survival curves were assessed by the Mantel-Cox test, and significance levels were corrected for multiple comparisons by the Bonferroni method. For these tests, *p< 0.0167.

## 3 Results

### 3.1 *In vitro* characterization of APC-targeting pDNA vaccines

To evaluate the efficacy of the APC-targeting strategy for the delivery of *in silico* predicted neoepitopes, we selected 11 APC-binding molecules reported to bind to receptors on the surface of cDCs. Specifically, we chose molecules binding receptors implicated in i) the endocytosis of antigens, ii) the regulation of the cDC migration, and iii) the maturation process of cDCs (**Supplementary Table 2**).

**Figure 1:**
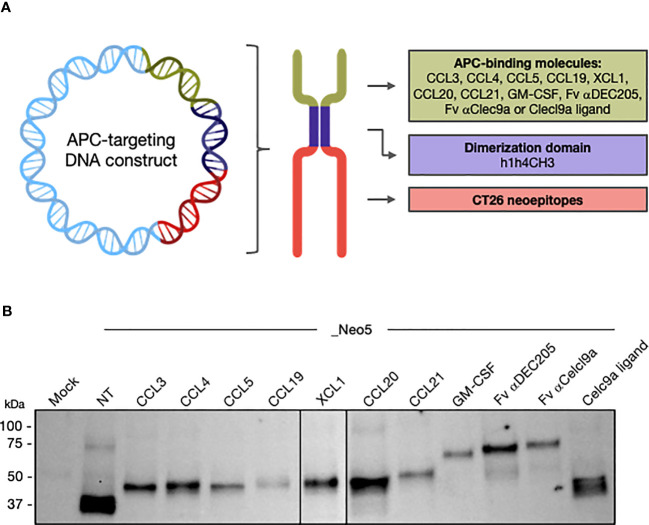
Design and *in vitro* characterization of APC-targeting pDNA constructs. **(A)** Schematic design of the pDNA construct and the encoded homodimeric fusion protein. Plasmid inserts containing the sequence of different APC-binding molecules, the dimerization module h1h4CH3, and 5 CT26-derived neoepitopes. GL linkers connect the three modules. The non-targeted control (NT_Neo5) and constructs harboring Fv αDEC205, Fv αClec9a, and Clec9a ligands had inserted secretion signals upstream the first coding element. Created with BioRender.com. **(B)** *In vitro* expression and molecular weight characterization of the APC-targeting fusion proteins by immunoblotting against the CH3 element in the supernatant of transfected HEK293T cells under reducing conditions. Representative of 3 independent experiments.

Using these molecules, we designed APC-targeting pDNA constructs encoding fusion proteins consisting of i) an APC-binding molecule, ii) the dimerization module h1h4CH3 from human IgG3, and iii) 5 *in silico* predicted CT26 neoepitopes previously published (33) (**Figure 1A**).

We confirmed that the different APC-targeting pDNA constructs resulted in the expression secretion of fusion proteins with the expected molecular weight in the supernatant of DNA-transfected HEK293T cells (**Figure 1B**). While acknowledging the limitations of immunoblotting for precise quantification, our data suggest potential differences in the expression levels of the vaccine candidates. Specifically, NT_Neo5, CCL20_Neo5, and Celc9a_Neo5 show the highest expression levels, while CCL19_Neo5 exhibits the lowest.

### 3.2 APC-binding molecules improve the efficacy of pDNA-encoded neoepitopes

We assessed the potential of the different neoepitope-encoding APC-targeting pDNA vaccines (APCt_Neo5) to enhance T-cell responses and tumor rejection in the BALB/c syngeneic model of colon carcinoma, CT26, in a prophylactic treatment setting. In two separate experiments, mice were immunized i.m. with 5 µg of research-grade APCt_Neo5 pDNA. The elicited T-cell responses and tumor-rejection efficacy were compared to the non-targeted pDNA construct NT_Neo5, which contains the same selection of neoepitopes but not an APC-binding molecule.

To evaluate the potential benefits of incorporating APC-binding molecules in the tumor-control capabilities of the delivered neoepitopes, we intentionally opted for a suboptimal dose of 5 µg of pDNA. The dose selection was informed by prior research demonstrating that 50 µg of pDNA vaccine encoding Neo5 leads to nearly complete tumor abrogation in the CT26 tumor model (33), limiting our ability to evaluate improvements in the vaccine design.

**Figure 2:**
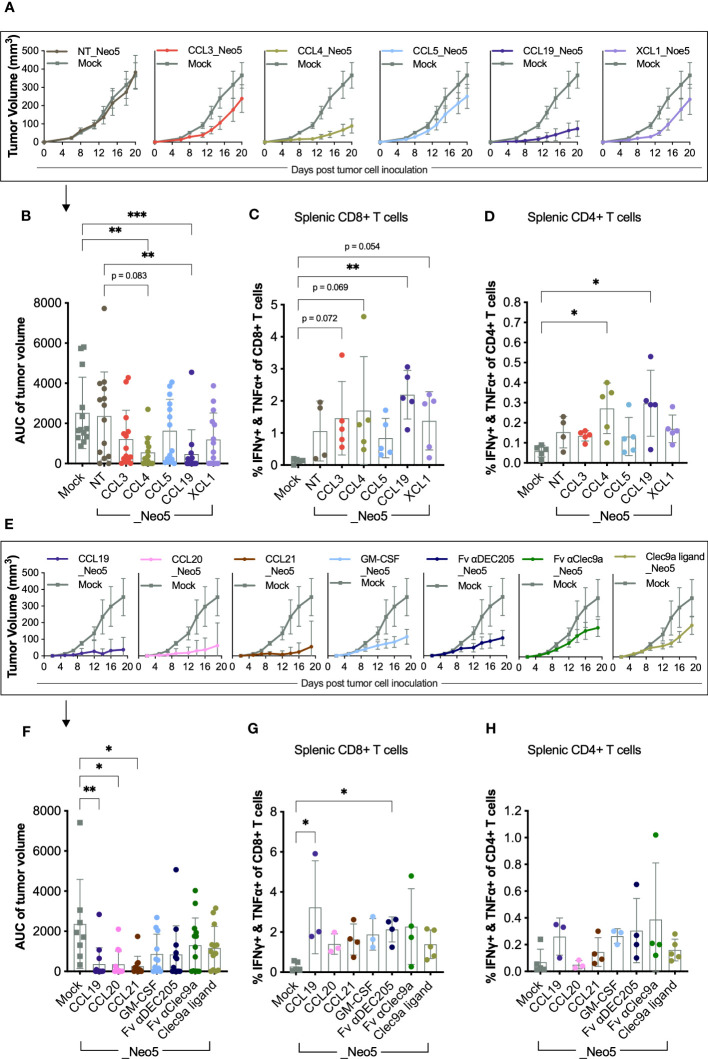
Tumor rejection and T-cell responses elicited by APC-targeting pDNA vaccines harboring different APC-binding molecules. BALB/c mice received five weekly immunizations of 5 μg pDNA in a prophylactic setup. Two weeks after the first immunization, mice were inoculated with 5 x 10^5^ CT26 tumor cells s.c. in the right flank (n =13 mice per group). The studies were terminated 20 days after tumor inoculation, and the spleen compartments were analyzed for TNFα and IFNγ-producing CD8+ and CD4+ T cells by ICS (n = 3-5 mice per group). The complete gating strategy used to identify specific the T-cell subsets is exemplified in **Supplementary Figure 7B**. **(A, E)** Mean tumor volume (mm^3^) ± SEM over time for each treatment group with LOCF. **(B, F)** Area under the curve (AUC) of individual tumors split by group. Mean ± SD. **(C, G)** Frequency of reactive splenic CD8+ T-cell and **(D, H)** CD4+ T-cells. Mean ± SD. Statistics: **(B–D)** APC_Neo5 groups were compared to the Mock DNA and NT_Neo5 groups by Kruskal-Wallis test and Dunn´s multiple comparison test. **(F–H)** APC_Neo5 groups were compared to Mock DNA group groups by Kruskal-Wallis test and Dunn´s multiple comparison test. Only comparisons where p-val< 0.1 are display in the figures. *p< 0.05, **p< 0.01, ***p< 0.001.

In the first experiment, we screened APCt_Neo5 pDNA constructs with the following APC-binding molecules: CCL3, CCL4, CCL5, XCL1, and CCL19. As expected, NT_Neo5 failed to induce significant tumor rejection (**Figures 2A, B**). However, it elicited immunogenicity, as evidenced by the presence of neoepitope-reactive T cells in the spleen compartment (**Figures 2C, D**). On the contrary, most mice receiving an APC-targeting vaccine developed smaller tumors than the mock control, which received an empty DNA plasmid (**Figures 2A, B**). The constructs harboring CCL4 and CCL19 as APC-binding molecules were the most efficient, with CCL19_Neo5 leading to statistically significant tumor control compared to the non-targeted construct NT_Neo5. Efficient tumor control correlated with the induction of high frequencies of neoepitope-reactive T cells measured by the presence of T cells that simultaneously produce TNFα and IFNγ upon neopeptide re-stimulation. APCt_Neo5 pDNA encoding CCL4 and CCL19 induced in average twice as many neoepitope-reactive CD8+ (2.43% and 2.19% respectively) and CD4+ (0.27% and 0.29% respectively) T cells compared to NT_Neo5 (1.06% CD8+ and 0.15% CD4+ reactive T cells) (**Figures 2C, D**). However, given the relatively high standard deviation in these parameters, the results need to be interpreted with caution.

Tumor control, and induction of reactive CD8+ and CD4+ T cells, are among the main readouts for determining the efficacy of TCVs. We ranked the different DNA constructs according to these three parameters to evaluate the performance of the different APC-binding molecules. The results designated CCL19 as the most promising APC-binding molecule for delivering *in silico*-predicted cancer neoepitopes, closely followed by CCL4 (**Supplementary Table 3** top).

In a subsequent experiment, we compared the efficacy of CCL19, CCL20, CCL21, GM-CSF, Fv αDEC205, Fv αClec9a, and Clec9a ligand as APC-binding molecules. All APC-targeting pDNA vaccines conferred superior tumor control compared to the mock control (**Figures 2E, F**) with CCL19, CCL20, and CCL21_Neo5 displaying statistically significant improvements. CCL19_Neo5 induced the highest levels of neoepitope-reactive reactive CD8+ and CD4+ T cells (3.23% and 0.26%, respectively) (**Figures 2G, H**) in the spleen compartment at the termination of the study. The ranking of the APC-binding molecules tested in the second experiment based on improved tumor control, and induction of reactive CD8+ and CD4+ T cells, pointed to CCL19_Neo5 as the most efficient APC-targeting construct (**Supplementary Table 3** bottom).

Despite the potentially lower expression levels, CCL19 performed as the best APC-binding molecule when combined with 5 CT26-predicted neoepitopes and, therefore, was selected to further develop an APC-targeted TCV for cancer neoepitopes.

### 3.3 Impact of the secretion signal and the covalent link between CCL19 and the neoepitopes in the efficacy of an APC-targeting pDNA vaccine

It is believed that, upon secretion of the APC-targeting construct, the APC-binding molecules interact with receptors in the surfaces of APCs, facilitating antigen internalization and presentation in MHC molecules and enhancing the immunogenicity of the antigens. This mechanism is possible because a covalent link connects both elements. In addition, APC-binding molecules such as chemokines can act as genetic adjuvants, modulating APC activation and recruitment to the vaccination site -processes independent of the covalent connection between the two elements-.

**Figure 3:**
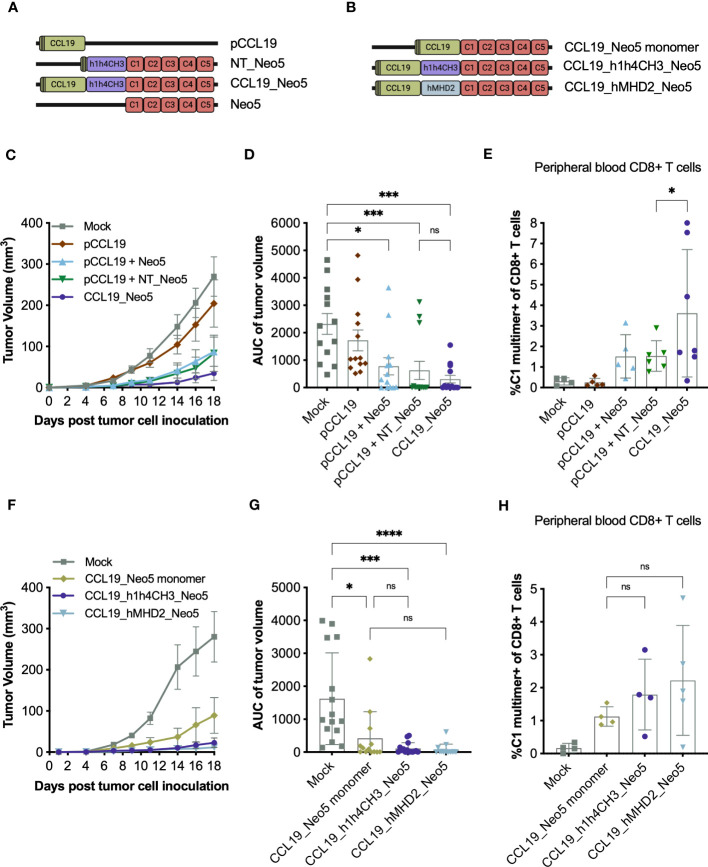
Contribution of the secretion signal, the covalent link between CCL19 and the neoepitopes, and dimerization module to the efficacy of APC-targeting pDNA vaccines. BALB/c mice received five weekly pDNA immunizations in a prophylactic setup. Seven or nine days after the first immunization, tail-vein blood was collected and stained with a neoepitope-specific MHC-I multimer to analyze the frequency of C1-specific CD8+ T cells in circulation (n = 4-7 mice per group). The complete gating strategy used to identify the CD8+ T-cell subset is exemplified in **Supplementary Figure 7A**. Two weeks after the first immunization, mice were inoculated with 5 x 10^5^ CT26 tumor cells s.c. in the right flank (n =13 mice per group). The studies were terminated 18 days after tumor inoculation. In the first experiment **(C–E)** mice received: i) 5 μg CCL19_Neo5, ii) 5 μg NT_Neo5 in combination with 5 μg pCCL19, iii) 5 μg Neo5 in combination with 5 μg pCCL19, iv) 5 μg CCL19 or v) 10 μg of Mock pDNA per immunization. In the second experiment **(F–H)** mice received 5 μg pDNA per immunization. **(A, B)** Schematic design of pDNA constructs. Created with BioRender.com. **(C, F)** Mean tumor volume (mm^3^) ± SEM over time for each treatment group with LOCF. **(D, G)** Area under the curve (AUC) of individual tumor volume split by group. Mean ± SD **(E, H)** % of C1-specific CD8+ T cells in circulation 9 and 7 days after the first immunization, respectively. Mean ± SD. Statistics: **(D, G)** Kruskal-Wallis test and Dunn´s multiple comparison test. **(E, H)** One-way ANOVA and Šidák´s multiple comparison test. All the comparisons performed are displayed in the figures. ns: p ≥ 0.05, *p< 0.05, ***p< 0.001, ****p< 0.0001.

To address the implications of the covalent link between the APC-binding molecule CCL19 and the cancer neoepitopes in CCL19_Neo5, we designed a tumor experiment comparing the efficacy of immunizing with the fusion construct CCL19_Neo5 or with the combination of NT_Neo5 and a pDNA encoding CCL19 (pCCL19) (**Figure 3A**). In addition, we evaluated the contribution of the secretion signal by comparing the efficacy of co-delivering pCCL19 and NT_Neo5 and co-delivering pCCL19 and Neo5, which contains the same neoepitopes but not a secretion signal (33).

Co-administration of pCCL19 and NT_Neo5 and treatment with CCL19_Neo5 led to complete tumor prevention in most animals, hampering the analysis of the potential impact of the link between CCL19 and the neoepitopes in preventing tumor development. Similarly, co-delivery of pCCL19 and Neo5 achieved tumor control in most of the animals (**Figures 3C, D**). These data suggest that neither the secretion signal nor the covalent link between CCL19 and the neoepitopes are essential for the tumor control capabilities of the CCL19_Neo5 pDNA vaccine. Nevertheless, given the limitations of the current setup, the results do not rule out the influence of these two factors in tumor prevention.

MHC-I multimer staining of the blood three weeks after the first immunization showed that immunization with CCL19_Neo5, which harbors a covalent link connecting CCL19 and the cancer neoepitopes, led to an average of 3,61% C1-specific CD8+ T cells, a significant increase when compared to the levels obtained by the co-administration of pCCL19 and NT_Neo5 (**Figure 3E**). On the other hand, introducing a secretion signal does not affect the levels of neoepitope-specific CD8+ T cells, with both co-administration regimes rendering ~1.5% C1-specific CD8+ T cells.

Analysis of the functional T-cell responses upon the study’s termination showed comparable levels of cytokine-secreting CD8+ (~3.5%) and CD4+ (~0.2%) T cells between treatment with CCL19_Neo5, co-administration of pCCL19 and NT_Neo5 and co-administration of pCCL19 and Neo5 (**Supplementary Figures 1A, B**).

Together the data suggest that including a secretion signal to pDNA-encoded neoepitopes does not impact the vaccine’s efficacy, while the introduction of a covalent connection between CCL19 and the cancer neoepitopes might favor early induction of C1-specific CD8+ T cells. Nevertheless, the benefit of the covalent link is restricted to the onset of the immune response as analysis of the spleen compartment as the end of the study show similar immunogenicity for the APC-targeting vaccine and the co-delivery regime. Administration of pCCL19 alone did not induce tumor protection or specific T-cell responses, underlying the neoepitope-dependency of the results.

### 3.4 Impact of the dimerization domain in the efficacy of an APC-targeting pDNA vaccine

Next, we evaluated the role of the dimerization module h1h4CH3 in the CCL19_Neo5 pDNA construct for which we generated the monomeric pDNA construct CCL19_Neo5 monomer and the homodimeric construct CCL19_hMDH2_Neo5, where the dimerization sequence hMDH2 substitutes h1h4CH3 (**Figure 3B**). Replacing h1h4CH3 with hMDH2 did not reduce the efficacy of the APC-targeted neoepitope vaccine, as both constructs achieved complete tumor rejection in most animals (**Figures 3F, G**). Removing the dimerization unit (i.e., CCL19_Neo5 monomer) also resulted in good tumor rejection, with no significant differences observed when comparing this group to those immunized with dimeric constructs. With the current setup, we are unable to scrutinize in depth the potential impact of substituting or eliminating the h1h4CH3 dimerization domain.

When analyzing the presence of neoepitope-specific T cells in circulation three weeks after the first immunization, we observed that CL19_h1h4CH3_Neo5, and CCL19_hMHD2_Neo5 induced on average twice as high frequencies of C1-specific CD8+ T cells (1.79% and 2.22%) than the monomeric construct CCL19_Neo5 monomer (1.25%) (**Figure 3H**). However, no statistically significant differences could be measured due to the limited group size and the high variation of the data set. Analysis of the spleen compartment indicated similar levels of neoepitope-reactive T cells between CL19_h1h4CH3_Neo5, and CCL19_ Neo5 monomer with the group receiving CCL19_hMHD2_Neo5 displaying the highest levels of reactive T cells (**Supplementary Figure 1C**). The changes in the dimerization domain did not affect the expression levels of the plasmids (**Supplementary Figure 1D**).

Together, the data indicate that in the current setup, including a dimerization module, regardless of its nature, has no significant impact on the anti-tumor efficacy of APC-targeting vaccines. Still, it could contribute to an earlier onset of the specific immune response.

### 3.5 APC-targeting of DNA-encoded neoepitopes increases vaccine efficacy five-fold and induces durable immunity

To bridge our preclinical results to a clinical setting for pDNA TCVs, we upgraded the backbone plasmid in CCL19_Neo5 from pUMVC4a to the more immunogenic pTVG4 (containing additional CpG motifs). The change in backbone plasmid did not alter the tumor control capability, which remained comparable for both pDNA designs (**Supplementary Figure 2A**). But we observed an improvement in the frequencies of C1 neoepitope-specific and cytokine-producing T-cell in the pTVG4-based construct (**Supplementary Figures 2B–D**).

Previous studies have demonstrated that delivering 13 neoepitopes, compared to 5, can prime the T cells to recognize and attack more targets, increasing the likelihood of tumor recognition and rejection (33). Following this strategy, we designed a pTVG4-based APC-targeted DNA construct containing 13 27-mer neoepitopes predicted from the CT26 cell line, called CCL19_Neo13, and manufactured it at clinical grade.

Change of the backbone plasmid to pTVG4 results in higher levels of CCL19_Neo5 in the supernatant of pDNA HEK293T-transfected cells. On the contrary, the introduction of additional neoepitopes has a detrimental effect on the expression level of CCL19_Neo13. Nevertheless, the CCL19_Neo13 fusion protein detected by immunoblot against the CH3 part of the protein, presents the expected molecular size, indicating that the neoepitopes are fully transcribed (**Supplementary Figure 2E**).

**Figure 4:**
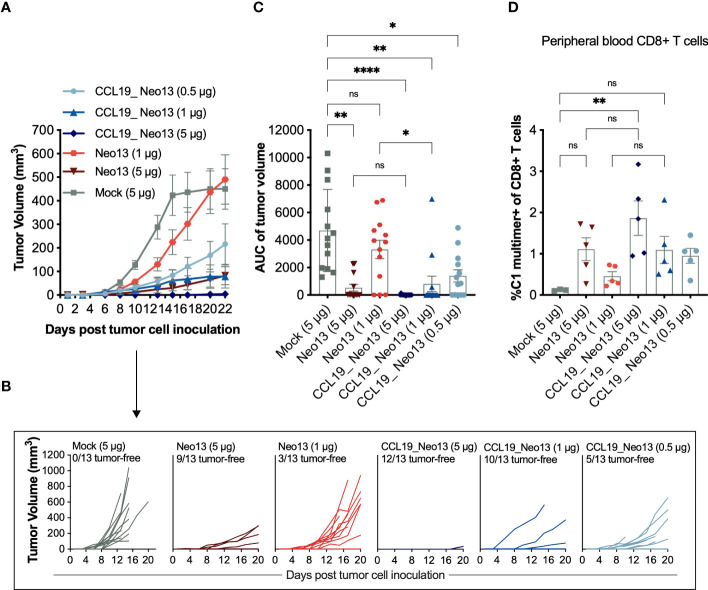
APC-targeting of pDNA-encoded neoepitopes elicits tumor prevention at low DNA doses. BALB/c mice received five weekly pDNA immunizations in a prophylactic setup. Sixteen days after the first immunization, tail-vein blood was collected and stained with a neoepitope-specific MHC-I multimer to analyze the frequency of C1-specific CD8+ T cells in circulation (n = 3-5 mice per group). The complete gating strategy used to identify the CD8+ T-cell subset is exemplified in **Supplementary Figure 7A**. Two weeks after the first immunization, mice were inoculated with 2,5 x 10^5^ CT26 tumor cells s.c. in the right flank (n =13 mice per group). The study was terminated 22 days after tumor inoculation. **(A)** Mean tumor volume (mm^3^) ± SEM over time for each treatment group with LOCF. **(B)** Tumor growth of individual mice over time. **(C)** The area under the curve (AUC) of individual tumors split by group. Mean ± SD **(D)** Frequency of C1-specific CD8+ T cells in circulation. Mean ± SD. Statistics: **(C)** Kruskal-Wallis test and Dunn´s multiple comparison test. **(D)** One-way ANOVA and Šidák´s multiple comparison test. All the comparisons performed are displayed in the figures. ns: p ≥ 0.05, *p< 0.05, **p< 0.01, ****p< 0.0001.

We evaluated the ability of the APC-targeted construct CCL19_Neo13 and the previously published, non-targeted pDNA design, Neo13 (33), to induce neoepitope-specific T cells and tumor control in a prophylactic setting at different pDNA doses. The results revealed a clear positive correlation between the dose of pDNA and the effectiveness of the treatment, determined by the frequency of circulating C1-specific CD8+ T cells and the ability to reject the tumors (**Figure 4**). Neo13 prevents tumor development at 5 μg of pDNA. The effect is lost when the pDNA dose is reduced to 1 μg. CCL19_Neo13 at 1 μg pDNA achieved significantly lower tumor volumes than the non-targeted construct. Notably, at the lowest tested dose, 0.5 µg of pDNA, CCL19_Neo5 retained partial tumor control when compared to the mock control, with five out of 13 animals remaining tumor-free upon the termination of the study (**Figures 4A–C**).

Overall, CCL19_Neo13 proved five times more potent than non-targeted version Neo13. 1 µg of CCL19_Neo13 resulted in 10 out of 13 tumor-free animals, comparable with the effect obtained with 5 µg of Neo13, where 9 out of 13 animals remained tumor-free. The frequency of C1-neoepitope-specific CD8+ T cells in circulation was comparable between the CCL19_Neo13 (1 µg) and Neo13 (5 µg) groups. CCL19_Neo13 at 0.5 µg retained immunogenicity evident by the presence of C1-neoepitope specific CD8+ T cells (**Figure 4D**).

In a separate experiment we interrogated the T-cell responses induced in the spleen compartment by 5 µg of CCL19_Neo13 and Neo13. We found significant differences in the frequencies of cytokine secreting CD8+ and CD4+ T cells between these two groups. CCL19_Neo13 induced an average of 2.5% and 0.36% reactive CD8+ and CD4+ T cells respectively, four times more than Neo13 (**Supplementary Figure 3**).

In a subsequent immunogenicity study, BALB/c mice were administered increasing doses of CCL19_Neo13, ranging from 5 to 500 µg of pDNA. At lower doses of CCL19_Neo13 pDNA, vaccine-induced T-cell responses correlate with the pDNA dose (**Supplementary Figure 4**). Thirteen days after the first immunization, 50 µg of pDNA vaccine induced over three times higher frequencies of C1-specific T-cell responses than 5 µg (**Supplementary Figure 3A**). The measured immune response plateaued at 50-100 µg, beyond which increasing the pDNA dose resulted only in marginal improvements in the frequencies of neoepitope-specific and reactive T cells.

To evaluate the duration of the vaccine-induced T-cell responses, we subjected groups of mice to one or four immunizations with 100 µg of CCL19_Neo13. C1-neoepitope-specific CD8+ T cells increased up to 20 days after the last immunization (study day 41) in both groups, representing 2.1% of the total CD8+ T cells in mice receiving four immunizations, and 1.2% in the group receiving one immunization. After study day 41, C1-neoepitope-specific T cells decreased over time, persisting at 0.8% and 0.28%, respectively, 105 days after the initiation of the study (**Supplementary Figure 5**).

### 3.6 APC-targeting neoepitope-based DNA vaccine elicits tumor control as monotherapy in an early-therapeutic setting

Next, we investigated if immunizing with CCL19_Neo13 post-tumor inoculation could achieve tumor control in the CT26 tumor model.

The fast kinetics of tumor growth in the syngeneic CT26 model provide a short time interval for evaluating the effectiveness of therapeutic vaccination regimes. To induce more rapid and robust cellular immune responses, we utilized EP-assisted pDNA vaccination. In a previous immunogenicity study, EP-assisted immunization with the CCL19_Neo13 DNA construct yielded stronger neoepitope-specific immune responses than poloxamer-formulated pDNA (**Supplementary Figure 6**).

**Figure 5:**
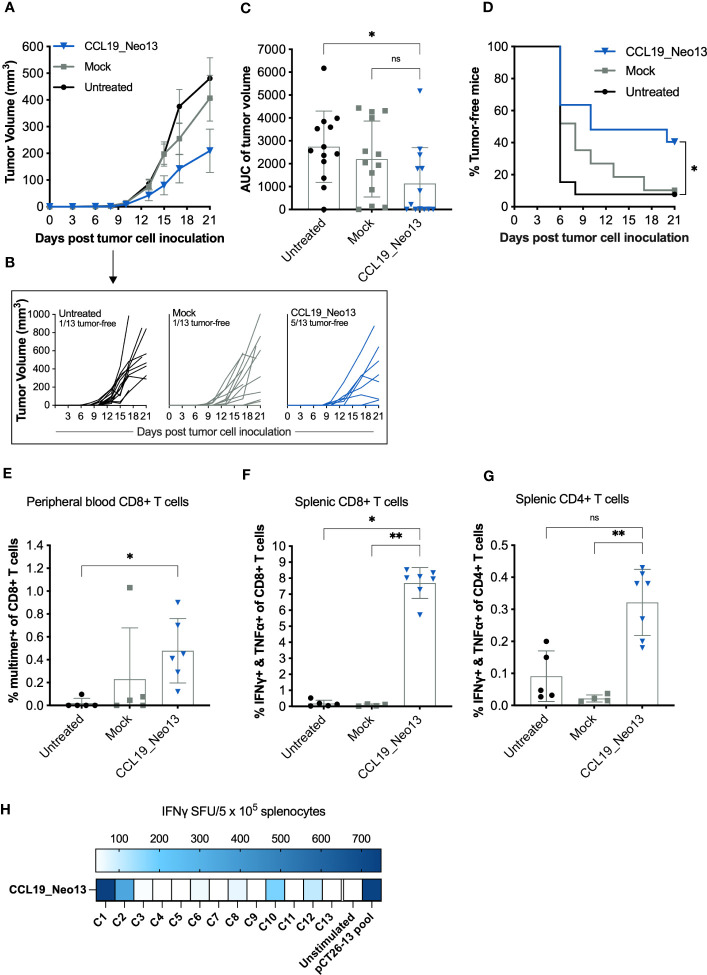
Neoepitope-encoding APC-targeting pDNA immunotherapy induces therapeutic tumor control. BALB/C mice were inoculated with 2 x 10^5^ CT26 tumor cells s.c. in the right flank (n =13 mice per group). One day after, the mice received five EP-assisted immunizations of 50 μg pDNA spaced over three to four days. Ten days after tumor inoculation, tail-vein blood was collected and stained with a neoepitope-specific MHC-I multimer to analyze the frequency of C1-specific CD8+ T cells in circulation (n = 5-6 mice per group). The study was terminated 21 days after tumor inoculation, and the spleen compartment was analyzed for TNFα and IFNγ-secreting CD8+ and CD4+ T cells by ICS (n = 4-7 mice per group). The complete gating strategies used to identify specific T-cell subsets is exemplified in **Supplementary Figure 7**. **(A)** Mean of group tumor volume (in mm^3^) ± SEM over time with LOCF. **(B)** Tumor growth of individual mice over time. **(C)** The area under the curve (AUC) of individual tumors split by group. Mean ± SD **(D)** Kaplan-Meier curve depicting % of tumor-free mice in each group over time. **(E)** Frequency of C1-specific CD8+ T in circulation. Mean ± SD. **(F)** Frequency of reactive CD8+ T cells and **(G)** CD4+ T cells. Mean ± SD. **(H)** IFNγ SFU/5x10^5^ splenocytes by ELISpot. Statistics: **(C, E–G)** Kruskal-Wallis and Dunn´s multiple comparison test. All the comparisons performed are displayed in the figures. ns: p ≥ 0.05, *p< 0.05, **p< 0.01 **(D)** Mantel-Coxt test with Bonferroni correction for multiple comparisons, *p< 0.0167.

To investigate the efficacy of APC-targeting DNA vaccines post-tumor inoculation, one day after s.c. inoculation of CT26 cells, mice received five EP-assisted immunizations of 50 µg of CCL19_Neo13 spaced two to three days apart. Mice immunized with the APC-targeting DNA vaccine exhibited partial tumor control with generally lower end-tumor volume (**Figures 5A–C**). Kaplan-Meier curves depicting tumor-free mice confirmed superior tumor control driven by CCL19_Neo13 with complete tumor abrogation in 5 out of 13 mice at the end of the study (**Figure 5D**). Multimer analysis of the blood nine days after the first immunization confirmed the presence of 0.5% C1-neoepitope-specific CD8+ T cells in mice receiving CCL19_Neo13 (**Figure 5E**). On day 21, upon the termination of the study, 8% of the CD8+ and 0.3% of the CD4+ T cells responded to neopeptide re-stimulation, producing both IFNγ and TNFα cytokines (**Figures 5F, G**). Further analysis of the neoepitope recognition profile of CCL19_Neo5 showed that T-cell reactivity is driven by several neoepitopes being C1, C2, C10 and C12 the predominant contributors to the vaccine-specific T-cell repertoire (**Figure 5H**).

## 4 Discussion

In this study, we assessed APC-targeting as a strategy to improve the immunogenicity and efficacy of a neoepitope-encoding pDNA vaccine. Our data demonstrate that including an APC-binding molecule to pDNA-encoded neoepitopes increases their immunogenicity and tumor control capabilities in the preclinical cancer mouse model CT26. Using the chemokine CCL19, we designed an APC-targeting pDNA vaccine, CCL19_Neo13, capable of inducing dose-dependent, robust, and long-lasting cellular immune responses, and anti-tumor efficacy. These data indicate that combining APC-targeting strategies with the delivery of personalized tumor antigens holds the potential to improve the efficacy of DNA TCVs in clinical settings.

Previous investigations of the mechanism of action of APC-targeted pDNA vaccines have established that targeting antigens to cDC receptors is an efficient way to induce strong cytotoxic T-cell responses (25). Targeting antigens to cDCs *in situ* promotes antigen internalization and processing, increasing MHC-presented epitopes’ availability and improving the priming of naïve T cells (22, 24). Following the modular APC-targeting pDNA design described by Fredriksen et al. (21), we tested 11 APC-binding molecules (CCL3, CCL4, CCL5, CCL19, CCL20, CCL21, XCL1, GM-CSF, Fv αDEC205, Fv αClec9a, and Clec9a ligand) for their ability to increase the immunogenicity and anti-tumor effect of five *in silico* predicted murine cancer neoepitopes. In the present study, most tested APC-targeting pDNA vaccines improve the anti-tumor efficacy compared to a non-targeted pDNA containing the same set of neoepitopes. Consistent with previous reports, we find a strong correlation between the induction of CD8+ and CD4+ reactive T cells by APC-targeted DNA vaccines and anti-tumor efficacy, indicating that neoepitope-induced T-cells mediate the anti-tumor effect (33). These results support the hypothesis that targeting neoepitopes to APCs is a powerful approach to enhance their immunogenicity and anti-tumor efficacy.

We selected CCL19 as APC binding molecule for further characterization based on its ability to increase the immunogenicity of the fused neoepitopes inducing a balanced neoepitope-specific CD4+ and CD8+ T-cell responses and leading to complete tumor rejection in a prophylactic setup. Despite inducing superior immunogenicity and anti-tumor effect, CCL19_Neo5 exhibited lower expression levels than other vaccine candidates, which could indicate that the nature of the APC-binding molecule has a more significant impact on the outcome of the vaccine than other parameters, such as the pDNA expression and secretion levels. However, it is important to note that the experimental approaches utilized here are not suited to quantify accurately the expression levels of the different vaccine candidates. Thus, we cannot draw definitive conclusions about the relationship between pDNA expression levels and vaccine performance. Similarly, the methodology used in this study does not allow us to evaluate the APC-binding molecules for their receptor-binding, internalization, or antigen presentation capabilities. Therefore, we will not discuss the implications of targeting specific receptors or defined DC subtypes in the immunogenicity and anti-tumor capabilities of APC-targeting pDNA vaccines.

When analyzing the contribution of the structural elements of CCL19_Neo5 to the immunogenicity and anti-tumor efficacy of the cancer neoepitopes, our data indicate that the elimination of the secretion signal does not influence the immunogenicity of the neoepitopes significantly. Nonetheless, in the current setup, we could not conclusively determine the impact of this element on the anti-tumor efficacy.

Similarly, we were unable to thoroughly investigate the role of the covalent link between CCL19 and the cancer neoepitopes as delivering them together -either fused in a single plasmid or two separate ones- effectively prevented tumor development. Alternatively, quantifying the neoepitope-specific T-cells shows the benefit of the covalent link between the two moieties. CCL19_Neo5 induces significantly higher frequencies of C1-specific CD8+ T cells in circulation than the co-administration of pCCL19 and NT_Neo5. However, the benefit of a covalent link seems to be restricted to the onset of the immune response, as their analysis in the spleen compartment shows no difference in the levels of specific IFNγ and TNFα-producing CD8+ and CD4+ T cells.

While the presented results provide valuable insights into the structural elements that influence the efficacy of APC-targeting pDNA vaccines, more research is needed to resolve the ongoing debate in the literature regarding the importance of a covalent link between the APC-binding molecule and the antigens. In accordance with our results, Westermann J. et al. conclude that the co-delivery of two different DNA plasmids encoding CCL19 and the immunogenic cancer antigen HER2 boosts immunogenicity and tumor protection (36, 37). These results acknowledge the role of CCL19 as a genetic adjuvant that could influence the immunogenicity of neoepitopes through additional mechanisms such as recruitment and activation of APCs to the immunization site. Others, however, find that the covalent link between the APC-binding molecule and the antigens is required to enhance antigen-specific T-cell responses and anti-tumor efficacy as co-delivery of both units in different DNA plasmids is insufficient for the vaccine’s success (22, 31). These discrepancies may be attributed to differences in the experimental design and the immunogenic characteristics of the antigens investigated.

Comparably to other reports (12), we find that the introduction of a dimerization module is not essential to enhance the immunogenicity of the encoded neoepitopes or to achieve an anti-tumor effect in the current setting, but it could contribute to an earlier onset of the adaptive immune response. Although the current group size and data variation limit our ability to draw definitive conclusions, the observed trends warrant deeper exploration to understand the potential advantages and mechanisms associated with different multimerization units. Rapid initiation of neoepitope-specific cytotoxic responses could be essential for rapid-growing or metastatic cancers where time is critical to increasing survival.

Expansion of the number of neoepitopes to 13 and upgrade of the backbone plasmid to pTVG4 resulted in the design of the pDNA vaccine candidate CCL19_Neo13. The long-lasting efficacy, of CCL19_Neo13 was evident in a longitudinal study assessing the induction of neoepitope-specific CD8+ T cells in circulation, which persisted for three months after the last immunization. The presented data agree with previous studies demonstrating the longevity of neoepitope-specific T-cell responses induced by pDNA vaccination (33).

We observed a strong correlation between the dose of CCL19_Neo13 pDNA and the strength of the T-cell responses and anti-tumor efficacy, corroborating the robustness of APC-targeting as a delivery platform for pDNA-encoded neoantigens. CCL19_Neo13 induced similar immune responses and tumor protection at five times lower doses than the non-targeted, non-secreted version Neo13. Furthermore, administration of CCL19_Neo13 post-tumor inoculation achieved a partial anti-tumor effect in the syngeneic tumor model CT26, marking a noteworthy stride in investigating the vaccine’s potential as adjuvant therapy and opening the door to explore it in combination with standard care treatments such as checkpoint inhibitors.

In conclusion, these studies asses APC-targeting as a strategy to improve the immunogenicity and efficacy of cancer neoepitope-containing pDNA vaccines. The results demonstrate that this approach is a powerful method to boost neoepitopes´ immunogenicity and anti-tumor efficacy in murine cancer models. Among the 11 APC-binding molecules tested, CCL19 was selected for further characterization due to its ability to induce strong and balanced neoepitope-specific T-cell responses, and render complete tumor rejection in a prophylactic setting and partial tumor control in an early-therapeutic setup.

The successful implementation of APC-targeting pDNA as a delivery platform for cancer neoepitopes represents an important contribution to the development of personalized TCVs. However, further research is needed to fully understand the implications of the specificity of APC-binding molecules on the immunogenicity and anti-tumor capabilities of APC-targeting DNA vaccines.

## Data availability statement

The original contributions presented in the study are included in the article/**Supplementary Material**. Further inquiries can be directed to the corresponding author.

## Ethics statement

The animal study was approved by The Danish Animal Experiments Inspectorate, Ministry of Food, Agriculture and Fisheries of Denmark. The study was conducted in accordance with the local legislation and institutional requirements.

## Author contributions

MB-C, BR, SF, JK and AS conceived the study. MB-C, SF, BR, JK, DK-K, CS, CT, and AS performed background research and designed the DNA constructs. MB-C, SF, BR planned the experiments. MB-C, SE, SK and NV conducted the experiments. MB-C, SK and SE analyzed the data. BR, SF, MB-C, SK, JK and AS interpreted the results. MB-C and SF wrote the first draft of the manuscript. MB-C, SF, SK, SE, NV, BR and AS contributed to the finalization of the manuscript. All authors contributed to the article and approved the submitted version.
