# Corrigendum: Targeting neoantigens to APC-surface molecules improves the immunogenicity and anti-tumor efficacy of a DNA cancer vaccine

**DOI:** 10.3389/fimmu.2023.1290431

**Published:** 2023-09-14

**Authors:** Marina Barrio-Calvo, Søren Vester Kofoed, Sofie Cens Holste, Anders Bundgård Sørensen, Nadia Viborg, Jens Vindahl Kringelum, Daniela Kleine-Kohlbrecher, Christian Skjødt Steenmans, Christian Bahne Thygesen, Birgitte Rønø, Stine Friis

**Affiliations:** Evaxion Biotech, Hørsholm, Denmark

**Keywords:** APC-targeting, CCL19, neoantigens, DNA vaccine, cancer immunotherapy

In the published article, there was an error in the Funding statement. The authors inadvertently omitted a funding disclosure that should have been included in the funding section of the manuscript. The correct Funding statement appears below.

