# Supplementary material for: Targeting neoantigens to APC-surface molecules improves the immunogenicity and anti-tumor efficacy of a DNA cancer vaccine
Source: Front Immunol. 2023 Aug 29;14:1234912. doi: 10.3389/fimmu.2023.1234912 (PMC10499626; doi:10.3389/fimmu.2023.1234912)
Supplement: Supplementary file 1 [file DataSheet_1.docx]

Supplementary Material

**Supplementary table 1.** DNA sequences of the APC binding molecules used in this study.

*Excel file*

**Supplementary table 2.** Comprehensive overview of the selected APC-binding molecules for the design of APC-targeting DNA vaccines and the role of its receptor activation in the biology of dendritic cells.

|  |  | **APC-binding molecule** | **Targeted receptors** | **Tissue specificity of receptor** | **Role of endogenous ligand-receptor activation in the biology of dendritic cells** |
| --- | --- | --- | --- | --- | --- |
| Cytokines | Chemokines | CCL3 | CCR1, CCR5 | Immature migratory cDC-1 | Activation and recruitment of DCs to peripheral tissue promoting APC-antigen encounter and maturation in response to acute inflammation (1). |
|  |  | CCL4 | CCR5 |  |  |
|  |  | CCL5 | CCR1, CCR3, CCR5 |  |  |
|  |  | CCL20 | CCR6 |  | Activation and recruitment of DC to the skin and mucosal surfaces under homeostatic and inflammatory conditions (1). |
|  |  | XCL1 | XCR1 | Tissue-resident cDC-1 | Activation and recruitment of CD8+ DCs to peripheral tissue promoting APC-antigen encounter (2). |
|  |  | CCL19 | CCR7 | Mature migratory cDC-1 | Migration of mature cDC-1 from peripheral tissues to lymphoid tissue (1). |
|  |  | CCL21 |  |  | Migration of mature cDC-1 within lymph nodes for co-location in the subcapsular sinus with antigen-specific T cells (1). |
|  | CSFs | GM-CSF | GM-CSF receptor | GM-CSFR | Regulation of homeostasis and development of precursors into common DC progenitors during DC development (3). |
| Engineered ligands | Fv fragments | Fv aDEC205 | DEC205 | Blood resident cDC-1 | Antigen endocytosis by blood resident cDC-1 under homeostasis and inflammatory conditions and cross-presentation to CD8+ T cells participating both in immune tolerance and inflammatory immune responses (4). |
|  |  | Fv aClecl9 | Clec9 | Tissue-resident cDC-1 | Recognition of necrotic cell death and mediation of cross-presentation of dead-cell associated antigens, especially during viral infection (1). |
|  | Small ligands | Clec9 pep ligand |  |  |  |

***S*upplementary table 3.** The rank of APC-targeting DNA constructs according to three different parameters contributing to the performance of the DNA vaccines: i) Anti-tumor effect depicted as the area under the curve (AUC) of individual tumor volumes over time, ii) Frequency IFNγ+ TNFα+ CD8+ T cells upon neopeptide restimulation and iii) Frequency of IFNγ+ TNFα+ CD4+ T cells upon neopeptide restimulation. Bold letters indicate the best performing construct for each evaluated parameter.

|  |  | **Tumor volume (AUC)** | | **% INFγ+ TNFα+ CD8+T cells** | | **% INFγ+ TNFα+ CD4+T cells** | |
| --- | --- | --- | --- | --- | --- | --- | --- |
| **Feature**  **DNA construct** | **APC-binding molecule** | Mean | SD | Mean | SD | Mean | SD |
| CCL19_Neo5 | CCL19 | **474.3** | **1205** | 2.194 | 0.759 | 0.297 | 0.164 |
| CCL4_Neo5 | CCL4 | 553.4 | 778.8 | **2.430** | **1.907** | **0.272** | **0.126** |
| XCL1_Neo5 | XCL1 | 1199 | 1321 | 1.380 | 0.909 | 0.170 | 0.069 |
| CCL3_Neo5 | CCL3 | 1220 | 1437 | 1.458 | 1.145 | 0.135 | 0.025 |
| CCL5_Neo5 | CCL5 | 1636 | 1562 | 0.842 | 0.612 | 0.131 | 0.095 |
| NT_Neo5 | - | 2371 | 2195 | 1.063 | 0.936 | 0.130 | 0.086 |
| Mock | - | 2522 | 1778 | 0.128 | 0.077 | 0.059 | 0.029 |
|  |  |  |  |  |  |  |  |
| CCL19_Neo5 | CCL19 | 361.1 | 813.3 | **3.237** | **2.318** | 0.260 | 0.139 |
| Fv αClec9_Neo5 | Fv αClec9 | 1307 | 1354 | 2.278 | 1.887 | **0.388** | **0.424** |
| Fv αDEC205_Neo5 | Fv αDEC205 | 845 | 1426 | 2.133 | 0.624 | 0.305 | 0.240 |
| GM-CSF_Neo5 | GM-CSF | 870 | 990 | 1.887 | 0.790 | 0.263 | 0.055 |
| CCL21_Neo5 | CCL21 | **267.4** | **487** | 1.630 | 0.772 | 0.145 | 0.107 |
| CCL20_Neo5 | CCL20 | 359.8 | 654.6 | 1.407 | 0.511 | 0.050 | 0.031 |
| Clec9 pep ligand_Neo5 | Clec9 ligand | 1168 | 1076 | 1.392 | 0.714 | 0.160 | 0.081 |
| Mock | Mock | 2367 | 2219 | 0.278 | 0.237 | 0.070 | 0.097 |

**References**

1. Lukacs-Kornek V, Engel D, Tacke F, Kurts C. The role of chemokines and their receptors in dendritic cell biology. *Front Biosci* (2008) 13:2238–2252. doi: 10.2741/2838/PDF

2. Kroczek RA, Henn V. The role of XCR1 and its ligand XCL1 in antigen cross-presentation by murine and human dendritic cells. *Front Immunol* (2012) 3:14. doi: 10.3389/FIMMU.2012.00014/BIBTEX

3. Ushach I, Zlotnik A. Biological role of granulocyte macrophage colony-stimulating factor (GM-CSF) and macrophage colony-stimulating factor (M-CSF) on cells of the myeloid lineage. *J Leukoc Biol* (2016) 100:481. doi: 10.1189/JLB.3RU0316-144R

4. Burgdorf S, Kurts C. Endocytosis mechanisms and the cell biology of antigen presentation. *Curr Opin Immunol* (2008) 20:89–95. doi: 10.1016/J.COI.2007.12.002
