# Supplementary material for: Targeting neoantigens to APC-surface molecules improves the immunogenicity and anti-tumor efficacy of a DNA cancer vaccine
Source: Front Immunol. 2023 Aug 29;14:1234912. doi: 10.3389/fimmu.2023.1234912 (PMC10499626; doi:10.3389/fimmu.2023.1234912)
Supplement: Supplementary file 2 [file DataSheet_2.docx]

Supplementary Material

**Supplementary figure 1. Effect of the covalent link between CCL19 and the neoepitopes and the dimerization module in the end-point immune response.** Bulk splenocytes were restimulated with neopeptides, and the immune response analyzed by (**A, B**) ICS to determine the frequency of TNFα and IFNγ secreting CD8+ and CD4+ T cells (n = 5-8 mice per group) and (**C**) ELISpot to determine the IFNγ SFU/5x10^5^ splenocytes (n = 5 mice per group). Mean ± SD. The complete gating strategy used to identify specific T-cell subsets is exemplified in Supplementary Figure 7B. (**D**) *In vitro* expression of the APC-targeting fusion proteins by sandwich ELISA against CCL19 in the supernatant of transfected CHO-K1 cells. Biological replicates. Statistics: One-way ANOVA and Šidák´s multiple comparison test. All the comparisons performed are displayed in the figures. ns: p ≥ 0.05.

**Supplementary figure 2. The immunogenic plasmid vector pTVG4 increases the immunogenicity of the delivered neoepitopes.** BALB/c mice received five weekly immunizations with 2 μg of DNA in a prophylactic setup. Fifteen days after the first immunization, tail-vein blood was collected and stained with a neoepitope-specific MHC-I multimer to analyze the frequency of C1-specific CD8+ T cells in circulation (n = 3-5 mice per group). Thirteen days after the first immunization, mice were inoculated with 2,5 x 10^5^ CT26 tumor cells s.c. in the right flank (n =13 mice per group). The study was terminated 17 days after tumor inoculation, and the spleen compartment was analyzed for TNFα and IFNγ-secreting CD8+ and CD4+ T cells by ICS*.* (**A)** Area under the curve (AUC) of individual tumors split by group. Mean ± SD. (**B**) Frequency of C1-specific CD8+ T cells in circulation. Mean ± SD. **(C)** Frequency of reactive CD8+ T cells. Mean ± SD. **(D)** Frequency of reactive CD4+ T cells. Mean ± SD. (**E)** *In vitro* expression and molecular weight characterization of the APC-targeting fusion proteins by immunoblotting against the CH3 part of the protein in the supernatant of transfected HEK293T cells under reducing conditions. The complete gating strategies used to identify specific T-cell subsets is exemplified in Supplementary Figure 7. Statistics: Kruskal-Wallis test and Dunn´s multiple comparison test. All the comparisons performed are displayed in the figures. ns: p ≥ 0.05, *p < 0.05, **p < 0.01.

**Supplementary figure 3. CCL19_Neo13 induces significantly higer immunogenicity than the non-targeted version Neo13.** BALB/c mice received five weekly pDNA immunizations of 5 μg in an immunogenicity setup. The study was terminated one week after the last immunization and the spleen compartment analyzed for **(A)**TNFα and IFNγ secreting CD8+ and **(B)** CD4+ T cells by ICS. Mean ± SD. The complete gating strategies used to identify specific T-cell subsets is exemplified in Supplementary Figure 7B. Statistics: One-way ANOVA and Šidák´s multiple comparison test. All the comparisons performed are displayed in the figures. *p < 0.05.

**Supplementary figure 4. Correlation of high f CCL19_Neo13 doses with enhanced immune response.** BALB/c mice received five weekly DNA immunizations (n = 6*-7* mice per group). Thirteen days after the first immunization, tail-vein blood was collected and stained with a neoepitope-specific MHC-I multimer to analyze the frequency of C1-specific CD8+ T cells in circulation (n = 6*-7* mice per group). The study was terminated 36 days after the first immunization, and the spleen compartment was analyzed for TNFα and IFNγ secreting CD8+ and CD4+ T cells by ICS (n = 6-7 mice per group). (**A**) Frequency of C1-specific CD8+ T cells in circulation. Mean ± SD **(B, C)** Frequencies of IFNγ and TNFα producing CD8+ and CD4+ T cells, respectively. Mean ± SD. The complete gating strategies used to identify specific T-cell subsets is exemplified in Supplementary Figure 7 Statistics: One-way ANOVA and Šidák´s multiple comparison test. All the comparisons performed are displayed in the figures. ns: p ≥ 0.05, *p < 0.05, **p < 0.01.

**Supplementary figure 5. Multiple immunizations with CCL19_Neo13 confer a durable immune response.** BALB/c mice received one or four immunizations of 100 μg of pDNA spaced by a week (n = 7 mice per group). At study days 0, 13, 27, 34, 41, 48, 63, 77, and 105, tail-vein blood was collected and stained with a neoepitope-specific MHC-I multimer to analyze the frequency of C1-specific CD8+ T cells in circulation (n = 6*-7* mice per group). (**A**) Longitudinal development of the frequency of C1-specific CD8+ T cells in circulation. Mean ± SD. Arrows under the x-axis indicate the time of pDNA immunization for each group. (**B**) Frequency of C1-specific CD8+ T cells in circulation at study day 41 and study day 105 respectively. Mean ± SD. The complete gating strategy used to identify specific neoepitope specific CD8+ T-cell is exemplified in Supplementary Figure 7A. Statistics: One-way ANOVA and Šidák´s multiple comparison test. All the comparisons performed are displayed in the figures. ns: p ≥ 0.05, *p < 0.05, **p < 0.01.

**Supplementary figure 6. Electroporation-assisted vaccination boosts the immunogenicity of CCL19_Neo13.** BALB/c mice received two weekly i.m. immunizations of 5 μg of CCL19_Neo13 pDNA formulated either with poloxamer 188 or in PBS and followed by electroporation (EP) (n = 4 mice per group). Fourteen days after the first immunization, tail-vein blood was collected and stained with a neoepitope-specific MHC-I multimer to analyze the frequency of C1-specific CD8+ T cells in circulation (n = 4 mice per group). The study was terminated 15 days after the first immunization, and the spleen compartment was analyzed for TNFα and IFNγ secreting CD8+ and CD4+ T cells by ICS (n = 4 mice per group). (**A**) Frequency of C1-specific CD8+ T cells in circulation. Mean ± SD **(B, C)** Frequencies of IFNγ and TNFα producing CD8+ and CD4+ T cells, respectively. Mean ± SD The complete gating strategy used to identify specific T-cell subsets is exemplified in Supplementary Figure 7. Statistics: One-way ANOVA and Šidák´s multiple comparison test. All the comparisons performed are displayed in the figures. ns: p ≥ 0.05, *p < 0.05, **p < 0.01, ***p < 0.001.

**Supplementary Figure 7. Gating strategies for identifying vaccine induced CD8+ and CD4+ T cells.** (**A**) Representative flow cytometry plots showing the gating strategy used to identify neoepitope specific T cells after tetramer staining of peripheral blood. After excluding debris and doublets based on forward scatter (FSC) and side scatter (SSC), CD3+ T cells were selected based on CD3+ expression. Within CD3+ gate, CD4+ and CD8+ T cells were distinguished based their respective markers. The percentage of C1-specific T cells in the CD8+ subset was determined using a fluorochrome-labeled MHC class I C1-specific tetramer. (**B**) Representative flow cytometry plots showing the gating strategy used to identify IFNγ and TNFα secreting T-cells after peptide re-stimulation and intracellular cytokine staining of splenocytes. After excluding debris and doublets based on forward scatter (FSC) and side scatter (SSC), live cells were gated for further analysis. CD3+ T cells were selected from the live cell population, and within the CD3+ gate, CD4+ and CD8+ T cells were distinguished based on CD4 and CD8 expression. The percentage of reactive T cells in each subset (CD4+ or CD8+) was determined using markers for the cytokines IFNγ and TNFα.
